# Supplementary material for: Absence of CCR2 Promotes Proliferation of Alveolar Macrophages That Control Lung Inflammation in Acute Respiratory Distress Syndrome in Mice
Source: Int J Mol Sci. 2022 Oct 26;23(21):12920. doi: 10.3390/ijms232112920 (PMC9659255; doi:10.3390/ijms232112920)
Supplement: Supplementary file 1 [file ijms-23-12920-s001.zip › ijms-1926525-Sup materials_Rev.pdf]

Supplementary Materials

Table S1. Antibodies for the myeloid panel for flow cytometry

	Laser	Fluorochrome	Marker	Antibody clone	Company
	BLUE	FITC	CD45	30-F11	Biolegend
	BLUE	PerCP-eFluor 710	CD103	2E7	eBioscience
	YG	PE-Cy7	CD11c	N418	Biolegend
	YG	PE	CD64	X54-5/7.1	Biolegend
	YG	PE-CF594	Siglec F	E50-2440	BD Biosciences
	RED	Alexa fluor 647	CD206	MMR	Biolegend
	RED	Alexa fluor 700	Ly6G	1A8	BD Biosciences
	RED	APC-Cy7	Ly6C	AL-21	BD Biosciences
	VIOLET	eFLUOR 450	CD11b	M1/70	eBioscience
	VIOLET	Horizon v500	MHCII	M5/114.15.2	BD Biosciences
Dump gate	VIOLET	BV650	CD3	17A2	Biolegend
Dump gate	VIOLET	BV650	CD19	6D5	Biolegend
Dump gate	VIOLET	BV650	NKp46	29A1.4	Biolegend
	UV	Zombie UV	Live/dead	-	Biolegend

Table S2. Antibodies for the iNOS panel for flow cytometry

Laser	Fluorochrome	Marker	Antibody clone	Company
BLUE	FITC	CD45	30-F11	Biolegend
YG	PerCP-Cy5.5	CD11c	N418	eBioscience
YG	PE-CF594	Siglec F	E50-2440	BD Biosciences
VIOLET	eFLUOR 450	CD11b	M1/70	eBioscience
UV	Zombie UV	Live dead	-	Biolegend
		Intracellular staining		
YG	PE-Cy7	iNOS	CXNFT	eBioscience

Table S3. Antibodies for the proliferation assay with Ki-67

Laser	Fluorochrome	Marker	Antibody clone	Company
BLUE	FITC	CD45	30-F11	Biolegend
YG	PE-Cy7	CD11c	N418	eBioscience
YG	PE-CF594	Siglec F	E50-2440	BD Biosciences
VIOLET	eFLUOR 450	CD11b	M1/70	eBioscience
UV	Zombie UV	Live dead	-	Biolegend
		Intracellular staining		
RED	Alexa fluor 647	Ki-67	16A8	Biolegend

Table S4. Antibodies for the proliferation assay with BrdU

Laser	Fluorochrome	Marker	Antibody clone	Company
BLUE	FITC	CD45	30-F11	Biolegend
YG	PE-Cy7	CD11c	N418	Biolegend
YG	PE-CF594	Siglec F	E50-2440	BD Biosciences
VIOLET	eFLUOR 450	CD11b	M1/70	eBioscience
UV	Zombie UV	Live dead	-	Biolegend
		Intracellular staining		
YG	PE	BrdU or Isotype control	-	BD Biosciences

Figure S1. Gating Strategies

Samples were analyzed in the Flow Jo V10 software and gated as follows.

Myeloid panel

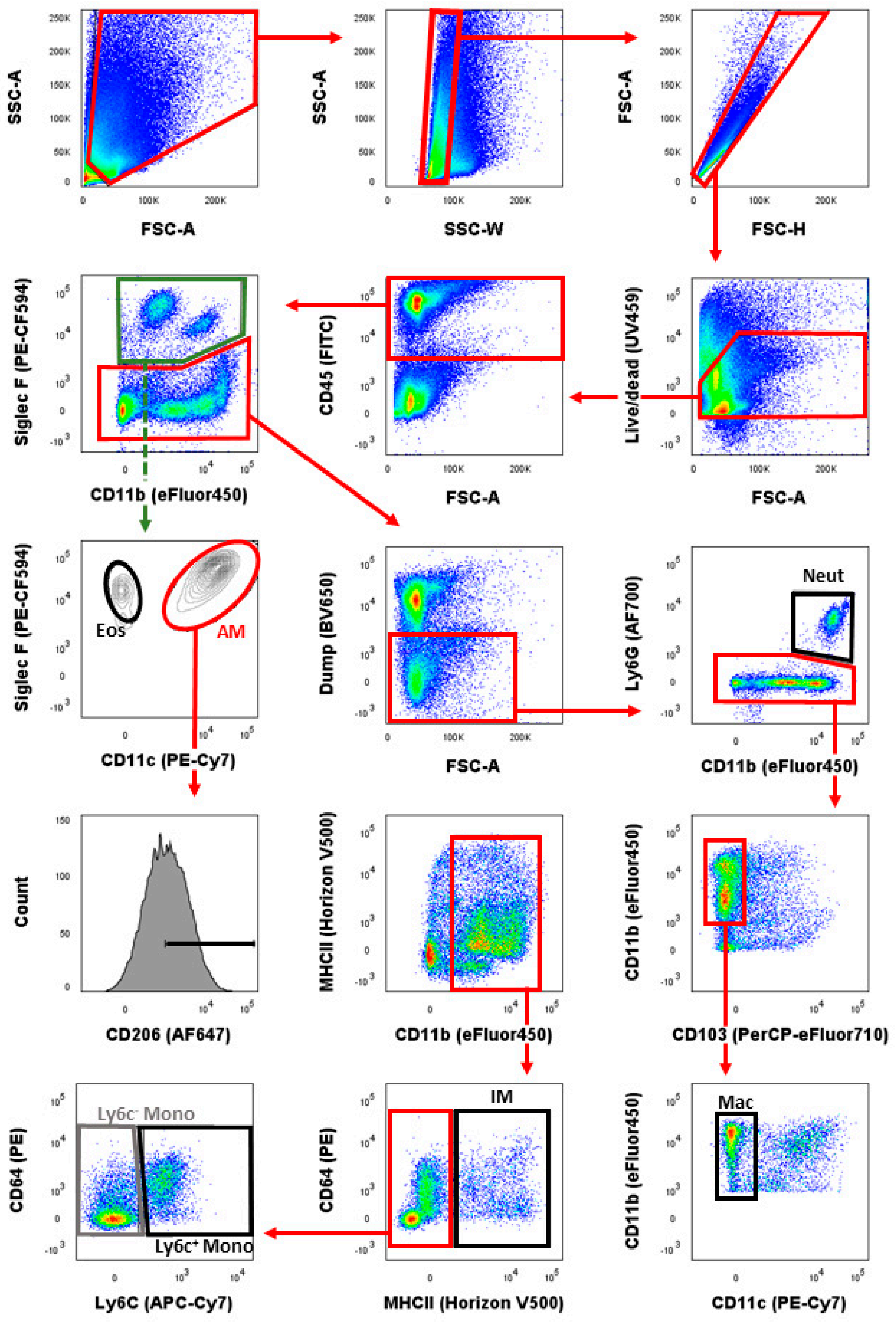

iNOS panel

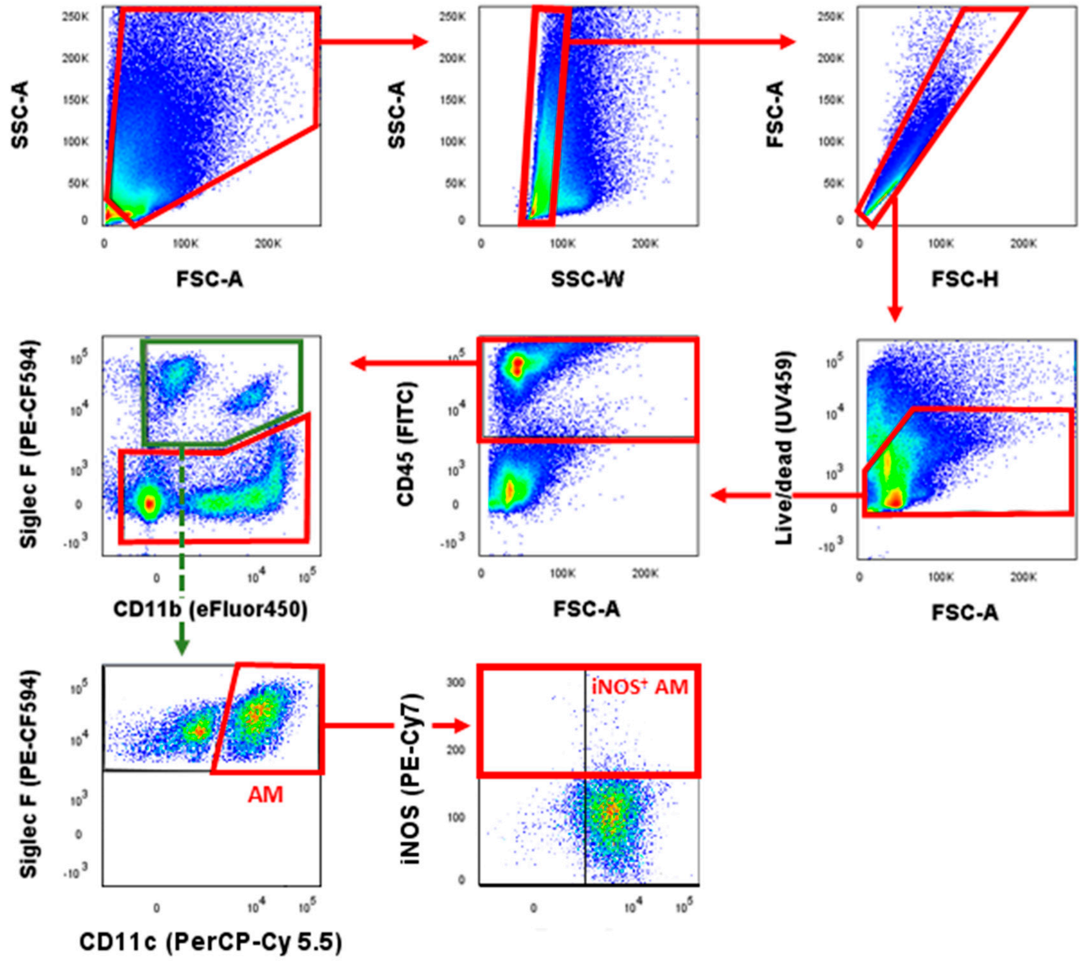

SSC-A
FSC-A
SSC-A
SSC-W
FSC-A
FSC-H
Siglec F (PE-CF594)
CD11b (eFluor450)
CD45 (FITC)
FSC-A
Live/dead (UV459)
FSC-A
Siglec F (PE-CF594)
CD11c (PerCP-Cy 5.5)
AM
iNOS (PE-Cy7)
iNOS+ AM

BrdU panel

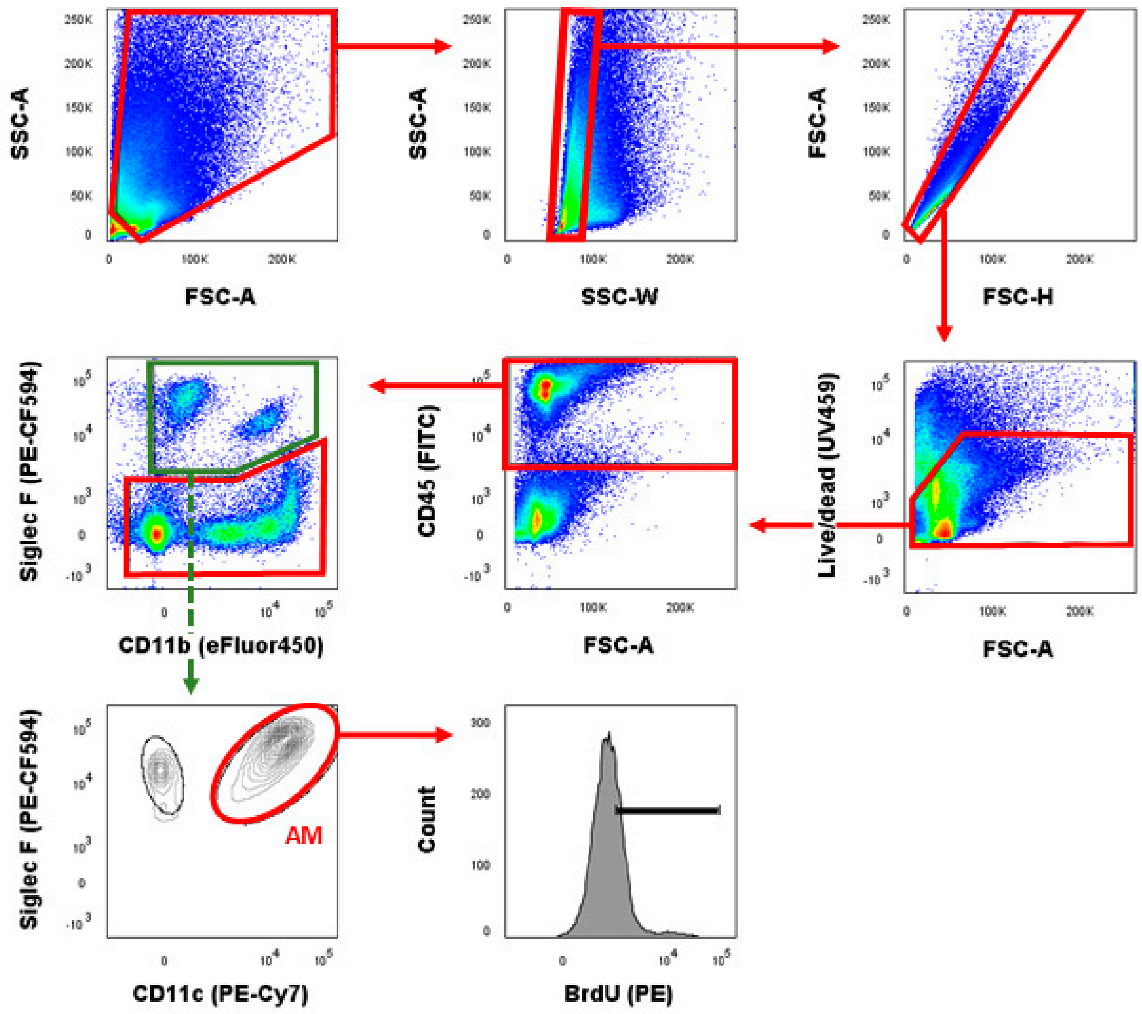
SSC-A
FSC-A
SSC-A
SSC-W
FSC-A
FSC-H
Live/dead (UV459)
FSC-A
CD45 (FITC)
FSC-A
Siglec F (PE-CF594)
CD11b (eFluor450)
Siglec F (PE-CF594)
CD11c (PE-Cy7)
AM
Count
BrdU (PE)

Ki-67 panel

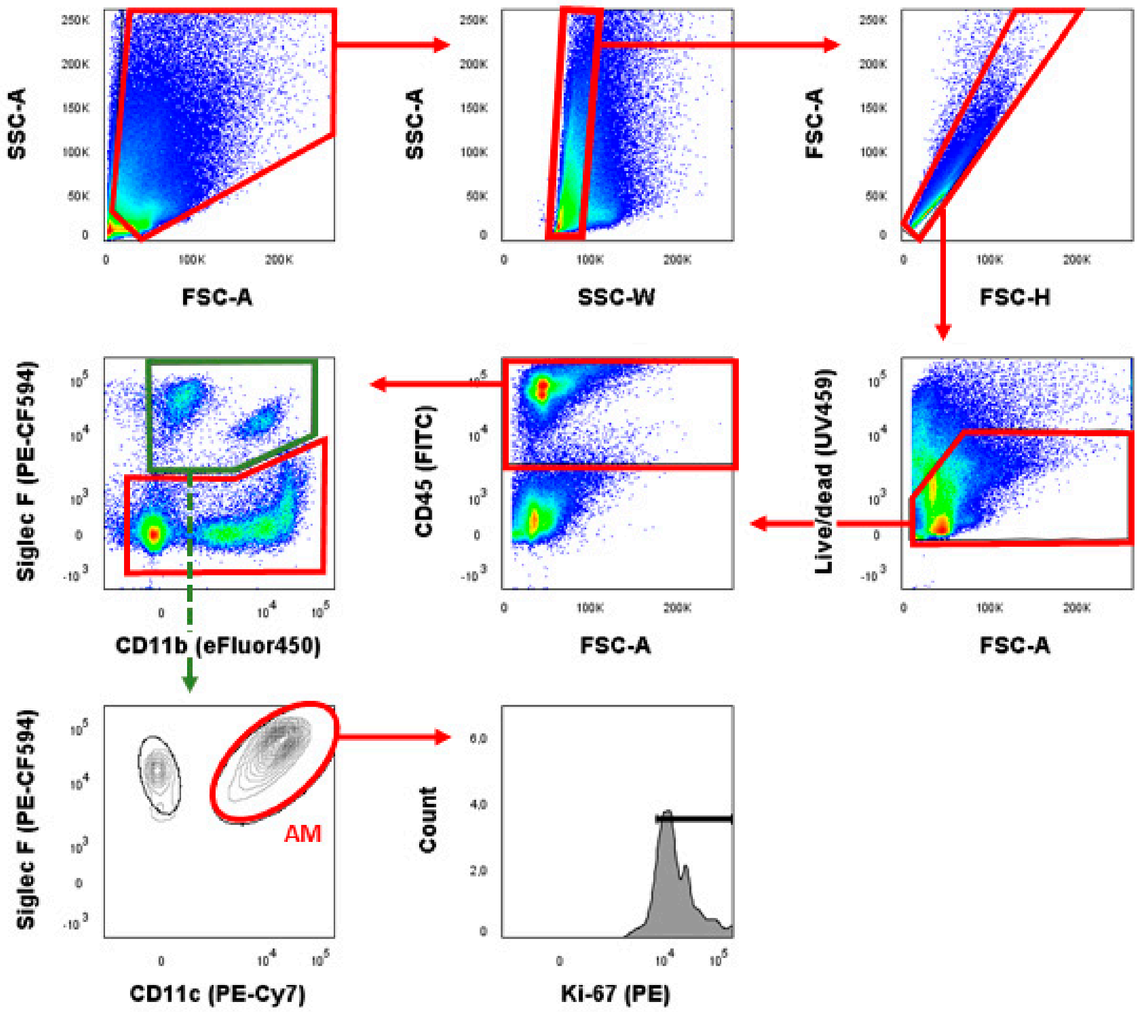

SSC-A
FSC-A
SSC-A
SSC-W
FSC-A
FSC-H
Siglec F (PE-CF594)
CD11b (eFluor450)
CD45 (FITC)
FSC-A
Live/dead (UV459)
FSC-A
Siglec F (PE-CF594)
CD11c (PE-Cy7)
AM
Count
Ki-67 (PE)

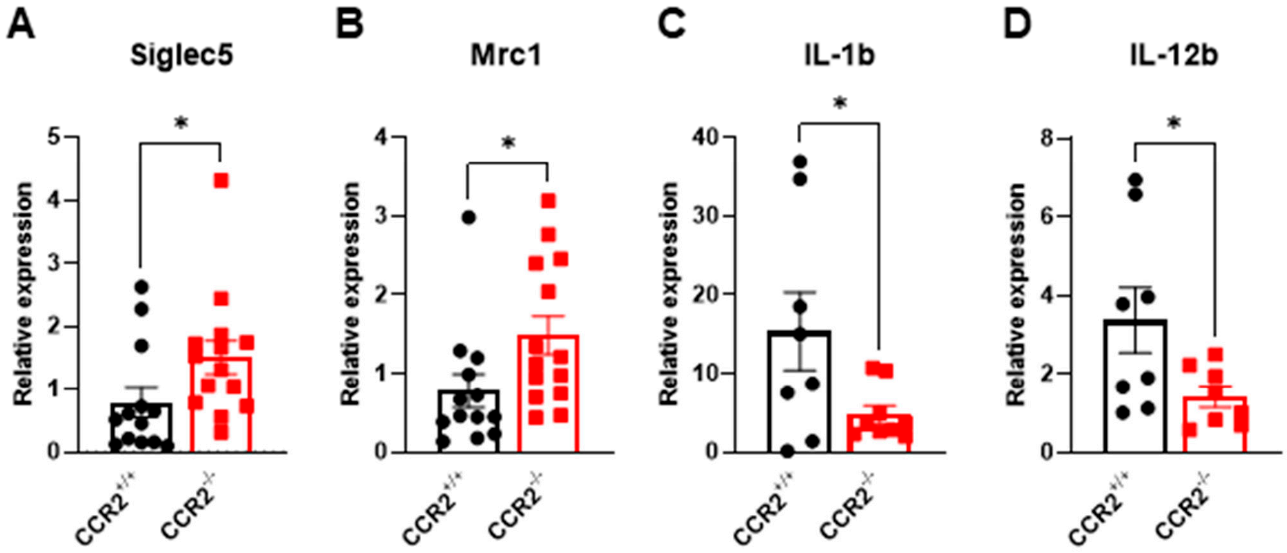

Supplementary figure S2. Expression of macrophage-associated genes in the lungs of CCR2$^{+/+}$ and CCR2$^{-/-}$ mice. CCR2$^{+/+}$ and CCR2$^{-/-}$ C57BL/6 mice were challenged with LPS (12.5 µg/mouse). 3 days post-instillation, the lungs were removed, and the expression of (A) Siglec5, (B) Mrc1, (C) IL-1b and (D) IL-12b was determined using qPCR. Results are represented as gene expression relative towards lungs that were not instilled with LPS. Statistical differences were determined using Mann-Whitney U tests (A-C) or unpaired t test (D) (*$p < 0.05$).

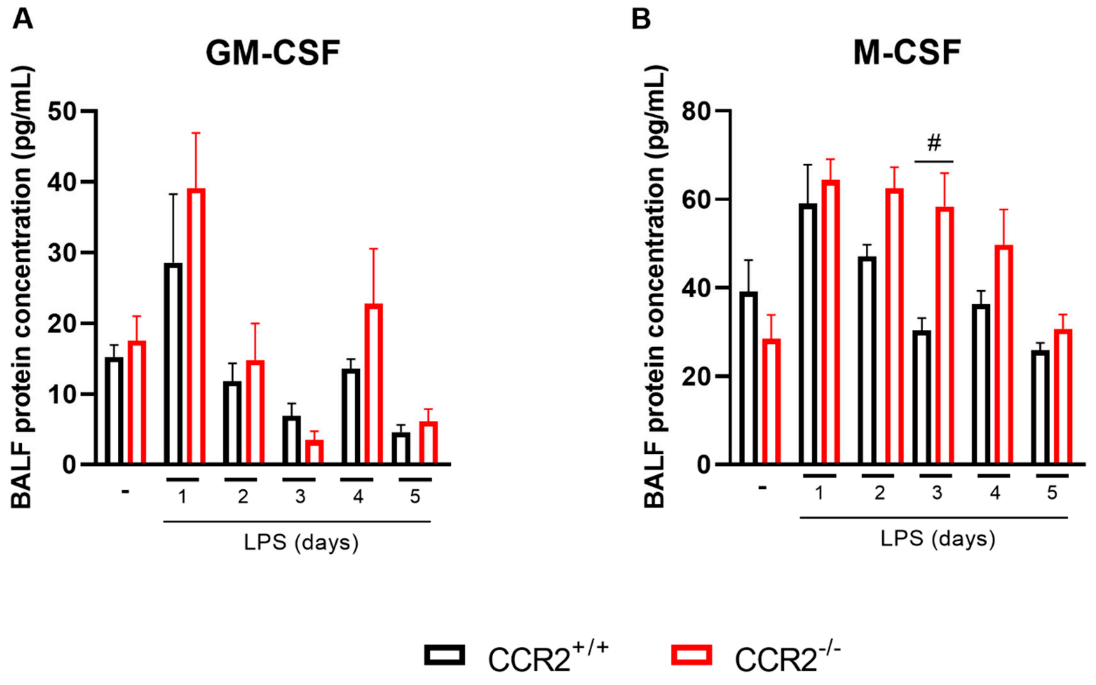

Supplementary figure S3. Levels of GM-CSF and M-CSF in CCR2$^{+/+}$ and CCR2$^{-/-}$ mice. CCR2$^{+/+}$ and CCR2$^{-/-}$ C57BL/6 mice were challenged with LPS (12.5 µg/mouse) or PBS (ctrl group) intranasally and dissected at the indicated days. Levels of GM-CSF (A), and M-CSF (B) measured in the BALF by ELISA. Data are shown as mean ± SEM. #$p<0.05$ when comparing wild type and knockout group at the same time point. ANOVA test followed by Bonferroni correction was used in panel B; Kruskal-Wallis with Dunn's multiple comparisons test was used in panel A. n=4-12.
